# Sex-differential RXRα gene methylation effects on mRNA and protein expression in umbilical cord of the offspring rat exposed to maternal obesity

**DOI:** 10.3389/fcell.2022.892315

**Published:** 2022-08-16

**Authors:** Erika Chavira-Suárez, Luis Antonio Reyes-Castro, Itzel Ivonn López-Tenorio, Lilia Vargas-Hernández, Guadalupe L. Rodríguez-González, Roberto Chavira, Paola Zárate-Segura, Aaron Domínguez-López, Felipe Vadillo-Ortega, Elena Zambrano

**Affiliations:** ^1^ Unidad de Vinculación Científica de la Facultad de Medicina, Universidad Nacional Autónoma de México en el Instituto Nacional de Medicina Genómica, Mexico City, México; ^2^ Departamento de Bioquímica, Facultad de Medicina, Universidad Nacional Autónoma de Mexico, Mexico City, México; ^3^ Departamento de Biología de la Reproducción, Instituto Nacional de Ciencias Médicas y Nutrición Salvador Zubirán, Mexico City, México; ^4^ Escuela Superior de Medicina, Instituto Politécnico Nacional, Mexico City, México; ^5^ Instituto Mexicano del Seguro Social, Hospital de Ginecología y Obstetricia No. 4 Luis Castelazo Ayala, Mexico City, México

**Keywords:** DNA methylation, sexual dimorphism, maternal diet, corticosterone, estradiol, steroid hormones, programming, fetal weight

## Abstract

Maternal obesity (MO) induces negative consequences in the offspring development. Adiposity phenotype is associated with maternal diet at early pregnancy and DNA methylation marks in the RXRα promotor at birth. Glucocorticoids play an important role in the regulation of metabolism through the activation of nuclear hormone receptors such as the RXRα protein. The aim of the study was to analyze steroid hormone changes at the end of pregnancy in the obese mother and RXRα gene methylation in the umbilical cord. For this purpose, in a well-established MO model, female Wistar rats were fed either standard chow (controls: C) or high-fat obesogenic diet (MO) before and during pregnancy to evaluate at 19 days of gestation (19 dG): 1) maternal concentration of circulating steroid hormones in MO and C groups, 2) maternal and fetal weights, 3) analysis of correlation between hormones concentration and maternal and fetal weights, 4) DNA methylation status of a single locus of RXRα gene near the early growth response (EGR-1) protein DNA binding site, and 5) RXRα mRNA and protein expressions in umbilical cords. Our results demonstrate that at 19 dG, MO body weight before and during pregnancy was higher than C; MO progesterone and corticosterone serum concentrations were higher and estradiol lower than C. There were not differences in fetal weight between male and female per group, therefore averaged data was used; MO fetal weight was lower than C. Positive correlations were found between progesterone and corticosterone with maternal weight, and estradiol with fetal weight, while negative correlation was observed between corticosterone and fetal weight. Additionally, male umbilical cords from MO were hypermethylated in RXRα gene compared to male C group, without differences in the female groups; mRNA and protein expression of RXRα were decreased in F1 male but not in female MO compared to C. In conclusion, MO results in dysregulation of circulating steroid hormones of the obese mothers and low fetal weight in the F1, modifying DNA methylation of RXRα gene as well as RXRα mRNA and protein expression in the umbilical cord in a sex-dependent manner.

## Introduction

The developmental programming concept (Barker, 2002; Barker, 2005), also known as developmental origins of health and disease (DOHaD), states that challenges in critical developmental time windows alter development with persisten effects on offspring phenotype (McMullen and Swali, 2013). Obesity is the most prevalent nutritional disorder in childhood (Fryar et al., 2020; World Health Organization, 2020). Multifactorial mechanisms such as parental nutrition and lifestyle, psychosocial and neuroendocrine status, genetic background, physical activity, adverse intrauterine environment during pregnancy, lacking breastfeeding, among others, contribute to obesity in children (Lau et al., 2011; Parlee and MacDougald, 2014; Pauwels et al., 2019; Bautista et al., 2021).

During pregnancy, maternal nutritional status is a crucial factor for modulating developmental programming in the offspring (Lau et al., 2011). Experimental evidence using hypo and hypercaloric maternal diets in animal models directly correlates with epigenetic changes in fetal tissues and various effects on the offspring phenotypes (Ganu et al., 2012), generating a great debate on the specific weight of epigenetic effects in the etiology of obesity.

In human pregnancy, obesity can result in abnormal fetal growth (Vieira et al., 2017). One biological mechanism thought to underlie this relationship is the fetal epigenetic programming (Saffery and Novakovic, 2014) by circulating steroid hormones stimuli (Socha et al., 2016; Houshdaran et al., 2020). The fetus must adapt to the supply of nutrients crossing the placenta, where peripheral endocrine regulation is a determinant for maternal metabolism coming into play for adequate fetal growth (Murphy et al., 2006). The placenta produces numerous hormones, including progesterone and estrogens, that work together to regulate maternal metabolism and may have essential participation in the regulation of fetal size (Mucci et al., 2003). Moreover, this transitory organ also maintains the glucocorticoid balance between the mother and the fetus.

Maternal obesity during pregnancy is associated with a pro-inflammatory intrauterine environment and lipotoxicity in the placenta, which has a link with adverse long-term metabolic programming in the offspring (Saben et al., 2014). Early growth response-1 (EGR-1) protein induces inflammatory cytokines expression in the trophoblast (Saben et al., 2013). Interestingly, the DNA-binding element for early growth response (EGR) proteins in the promoter region of the retinoid X receptor alpha (RXRα) gene is susceptible to differential DNA methylation (Chávez-Lizárraga et al., 2018).

DNA methylation is an epigenetic mechanism involved in the interaction between nutritional status and modulation of gene expression in individuals (Choi and Friso, 2010). During early human pregnancy, maternal carbohydrate intake proportion is linked with changes of DNA methylation of the RXRα gene promoter in the umbilical cord at birth, which is correlated with adiposity in children by age 9 years (Godfrey et al., 2011). Moreover, a maternal diet enriched with essential nutrients for fetal development inversely correlates with RXRα methylation in the placenta, which is associated with the newborn anthropometric characteristics (Nakanishi et al., 2021). RXRα protein belongs to the steroid and thyroid hormone receptor superfamily, acting as a transcriptional factor of genes linked to developmental biology and adipocytokine signaling pathway (Zhang et al., 2015). These results suggest that maternal diet, including nutrients rich in methyl groups during pregnancy, affects DNA methylation of key genes as RXRα in placenta and umbilical cord, contributing to developmental programming of the offspring (Burdge et al., 2007; Harvey et al., 2014; Nakanishi et al., 2021).

Steroid hormones during pregnancy play a critical role in the regulation of metabolism through their interaction with intracellular receptors. This includes RXRα protein that acts as transcriptional factor causing changes on gene expression (Evans and Mangelsdorf, 2014). In rodents, maternal serum corticosterone is considered the main glucocorticoid involved in regulating the stress response, having significant repercussions on developmental programming with sexual dimorphism (Zambrano et al., 2015; Rodríguez-González et al., 2019). Therefore, steroid hormones, including glucocorticoids, may have crucial contributions to fetal programming *via* modulation of epigenetic changes. Further study of transcriptional regulation mechanisms could provide evidence of molecular mechanisms involved in obesity-induced cellular and tissue dysfunctions in energy expenditure metabolism and programming of the offspring.

The rat model used in this study is a well-characterized method for exploring maternal steroid hormones concentration and epigenetic changes in the offspring as causal mechanisms in developmental programming influenced by maternal nutritional status (Zambrano et al., 2010; Rodríguez-González et al., 2015; Lomas-Soria et al., 2018). Epigenetic studies in the umbilical cord are important because, it is a tissue containing fetal vascular endothelial and mesenchymal stem cells with possible implications for future adiposity (Kadam et al., 2009). In addition, experimental evidence has been found that DNA methylation changes in the liver and heart induced by maternal diet are similar to those found in the umbilical cord (Lillycrop et al., 2005; Burdge et al., 2007).

In this study, we analyzed RXRα gene methylation (in the first exon near the response element of EGR-1) and mRNA and protein expression of RXRα in umbilical cords at 19 days of gestation offspring from obese rat mothers exposed to a high-fat diet. We examined maternal and fetal weights and their correlation, considering the low fetal weight in rodents as an adverse perinatal outcome associated with maternal obesity. Therefore, we hypothesized that obesity during pregnancy induces dysregulation in circulating steroid hormones of the obese mothers, disturbing DNA methylation of the RXRα gene and expression of RXRα mRNA and RXRα protein in umbilical cord of the offspring in a sex-dependent manner.

## Materials and methods

### Maternal obesity rat model

Experiments were conducted by following the principles of the Animal Experimentation Ethics Committee of the Instituto Nacional de Ciencias Médicas y Nutrición, Salvador Zubirán (INCMNSZ), Mexico City, Mexico (CINVA, institutional reference numbers: BRE-1868-16/19-1) and performed according to the Guidelines for the Care and Use of Laboratory Animals of the Institute of Laboratory Animal Resources (http://www.nal.usda.gov/awic/animal-welfare-act). Female albino Wistar rats were born and maintained in the INCMNSZ animal facility, accredited by and adhering to the Association for Assessment and Accreditation of Laboratory Animal Care International (AAALAC) standards.

For details in general procedures relating to the care of rats, maintenance, maternal diet, mating process, breeding, and management of control and obese mothers, consult the following references (Zambrano et al., 2010; Zambrano et al., 2021; Rodríguez-González et al., 2019). Briefly, at delivery on day 0, litters that provided founder generation (F0) mothers were culled to 10 pups, each containing at least four females. At weaning (day 21) one female F0 pup from each litter was randomly assigned to either a maternal control (C) group fed laboratory chow (LabRodent Diet 5001, Fort Worth, TX, United States that contains 23.9% protein, 5.0% fat, 31.9% polysaccharide, 23.2% simple sugars, 5.0% fiber, 7.0% minerals and ∼1.0% vitamins (w/w); energy provided = 3.4 kcal/g) or to a maternal obesity group (MO) fed a high energy, obesogenic diet (containing 22.5% protein, 20.0% animal lard, 5.0% fat, 20.5% polysaccharide, 20.5% simple sugars, 5.0% fibre, 5.0% mineral mix, 1.0% vitamin mix, (w/w); energy provided = 4.9 kcal g^−1^ (Rodríguez-González et al., 2019). There were not differences in F0 body weight at the initial of the study (21 d) among C and MO groups. The high-energy obesogenic diet was produced in the specialized dietary facility of the INCMNSZ. Thus, each F0 group had only one female from any litter, and F0 females in different groups, but not within groups were sisters, providing homogeneity in F0 mothers’ developmental programming and genetics. We report data with an *n* = 6 per F0 group.

**FIGURE 1:**
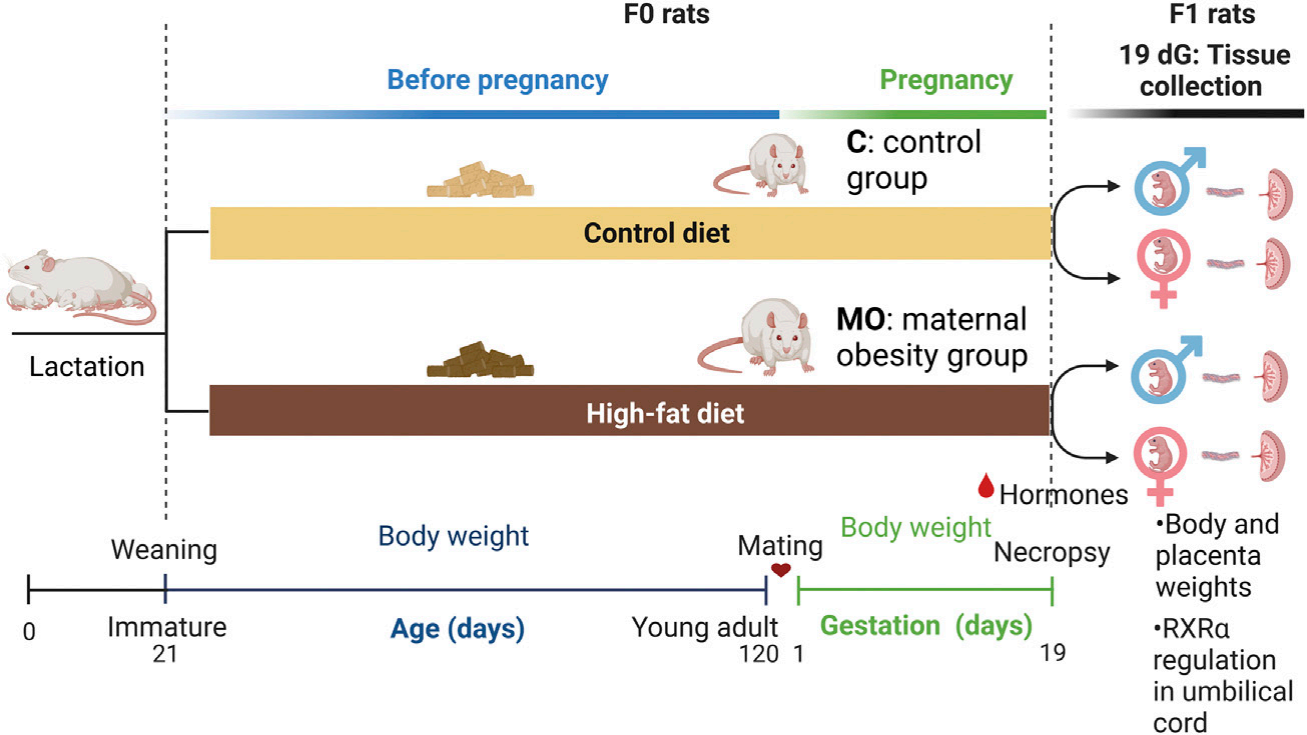
Maternal obesity study design. Experimental groups (*n* = 6 per maternal diet group), Female (F0) rats were fed with control (C) or high-fat (MO) diet before and during pregnancy and lactation. Mating around 120 days of age; dG, days of gestation; RXRα, retinoid X receptor alpha.

Body weight was measured weekly from weaning to 120 postnatal days (PND) in F0 MO and C groups. F0 young adult female rats were placed with proven male breeders following 120 PND and conceived during the next estrous cycle. Female rats were mated overnight with proven male breeders (Zambrano et al., 2020). The day on which spermatozoa were detected in a vaginal smear was designated as conception day 0. Fertility rate for C group was 75% and for MO group 25%. Throughout pregnancy, body weight (g) was recorded daily in both F0 groups until 19 dG, knowing that the average gestation time in the rat is 21 days (Figure 1).

### Steroid hormones quantification

At 19 dG, F0 rats were euthanized by decapitation with anesthesia, and blood samples took from the neck and were centrifuged at 3,500 rpm for 15 min at 4°C to remove red blood cells, and the serum was stored at −20°C until all samples were analyzed. Enzyme-linked immunosorbent assays (ELISAs) were performed, measuring samples by duplicate to quantified concentrations of serum hormones following manufacturer’s instructions for commercial rat kits. Estradiol (pg/ml) and corticosterone (ng/ml) concentrations were quantified using ELISA kits from DRG International, Inc. (EIA-4399 and EIA-5186, respectively; Springfield, NJ 07081 United States) and progesterone (ng/ml) and testosterone (ng/ml) concentrations were quantified using the kits from SIEMENS Immulite/Immulite 1000 Systems (LKPW1 and LKTW1, respectively; Llanberis, Gwynedd, LL55 4EL United Kingdom).

### Offspring litter measurements and tissue collection

At 19 dG, litter size, placenta and fetus (F1) weights as well as litter mass (total fetal and placenta weight) were recorded. The gonads of the fetus were observed with the support of a stereoscopic microscope by personnel trained to validate the sex and sex ratio per litter. Umbilical cord samples from the fetuses of each litter were collected in pools divided by sex and maternal diet group, preserving them in RNAlater (Invitrogen) following instructions of manufacture until their use.

### DNA and RNA extractions

Twenty-five milligrams of umbilical cord pooled from each litter were weighed and incubated in enzymatic tissue digestion solution [50 mM Tris-HCl, 100 mM EDTA, 100 mM NaCl, 1% SDS pH 8.0, and 0.5 mg/ml of proteinase K (Invitrogen)] at 50°C for 3 h, and then homogenized. DNA and RNA extraction of tissue samples was performed using 500 µl/sample of TRIzol reagent (Invitrogen) following manufacturers’ instructions. The nucleic acid quality was assessed by measuring the absorbance at 260/280 nm and was quantified with a Thermo Scientific Nanodrop 2000c Spectrophotometer (Thermo Scientific). The integrity of DNA and RNA was verified by 1% agarose gel electrophoresis and ethidium bromide staining. Isolated DNA and RNA samples were preserved at −76°C until examination.

### Primer designs and MS-HRM assay

Gene sequence of RXRα was obtained from the UCSC Genome Browser database on Rat July 2014 (RGSC 6.0/rn6) assembly. This location in the chr3:6,211,867-6,211,974 (107 pb) from the CpG 169 island corresponds to the first exon of the gene containing the response element [GCG(G/T)GGGCG (Lee, 2014)] for EGR-1 binding site in the rat genome. According to the MS-HRM technique conditions (Wojdacz and Dobrovic, 2007) the primers designed are the following: RXRα-F, 5′- GGG CGA GGG AGG GGG T -3′ and RXRα-R, 5′- CTA ACT CTC GAT ACC GCC AC-3′.

Five hundred milligrams of DNA were treated using the EZ DNA Methylation-Gold Kit (Zymo Research) following the manufacturer’s protocol. The recovered bisulfite-treated DNA was quantified in the spectrophotometer using the absorption coefficient at 260 nm.

MS-HRM conditions for the site-specific analysis of the RXRα gene were as follows: 1) for amplification, 10 min at 95°C followed by 45 cycles of 10 s at 95°C, 10 s at the primer annealing temperature (54°C), and 15 s at 72°C; 2) for high-resolution melting analysis, 1 min at 95°C, 5 s at 72°C, and continuous increase to 95°C with 50 acquisitions/°C; and 3), a cooling setting of 30 s at 40°C. MS-HRM assays were performed three times in duplicate white 96-well plates using a LightCycler 480 Instrument (Roche).

### Direct Sanger sequencing

Direct sequencing was performed in at least 10% of MS-HRM products in both alleles to validate RXRα methylation results. Previously, 500 ng of universal rat genomic DNA standards [high methylated control (HM) and low methylated control (LM) (EpigeneDx)] were treated using the EZ DNA Methylation-Gold Kit (Zymo Research) following the manufacturer’s protocol. 50 ng of treated DNA standards and MS-HRM products were re-amplified with the RXRα primers described above using a BigDye Terminator Kit and sequenced with an ABI prism 370 DNA sequencer (Applied Biosystems). The nucleotide sequences were aligned using Mega 6.06 and manually adjusted in the text editor. Initial identification of the sequences was made after performing BLAST searches of the NCBI database. The electropherogram quality was visualized with BioEdit Sequence Alignment Editor software v7.2.5, and CpG highlighting was simplified using the BiQ Analyzer software tool (Chávez-Lizárraga et al., 2018).

### Analysis of gene expression by RT-qPCR

Five micrograms of total RNA from umbilical cord samples were synthesized to cDNA using the Transcriptor First-Strand cDNA synthesis kit (Roche) following the manufacturer’s instructions. Assay efficiency was calculated using a dynamic range of cDNA dilution series (1:1, 1:2, 1:10, 1:100, 1:1,000, and 1:10,000). Quantitative real-time PCR was performed using the LightCycler TaqMan Master Kit (Roche), and TaqMan gene expression assay probes for RXRα and β-actin were purchased from Thermo Fisher Scientific. qPCR conditions for RXRα gene expression were the following: for amplification, 10 min at 95°C followed by 45 cycles of 10 s at 95°C, 30 s at the primer annealing temperature (60°C), and 10 s at 72°C. qPCR assays were performed three times in duplicate using a LightCycler Nano Instrument (Roche). Gene expression data were normalized using β-actin expression. Fold change in expression was calculated using the 2^−ΔΔCt^ method.

### Protein extraction and Western blotting

Fifty mg of umbilical cord sample pools from litters per sex were pre-treated with lysis buffer [50 mM Tris–HCl, 1% Nonidet P-40, protease inhibitor cocktail, pH 7.4 (Sigma Aldrich)] at 37°C for 24 h. The next day, samples were incubated in ice-cold lysis buffer for 1 h and homogenized. Homogenized samples were centrifuged at 13,000 rpm at 4°C for 5 min, and the supernatant was obtained and protein quantified by Bradford assay (Bio-Rad). Extracts were preserved at −76°C until their examination.

Fifty micrograms of protein in Laemmli buffer (1:1) were loaded in 15% SDS-PAGE gels and transferred to a polyvinylidene difluoride membrane (Thermo Scientific Pierce). The membrane was blocked with 5% non-fat dry milk dissolved in TBST for 30 min at room temperature. Blots were incubated with rabbit anti-RXRα [1:2,000 (Abcam)] or rabbit anti-β actin [1:2,000 (Abcam)] overnight at 4°C, washed with TBST, and incubated with secondary goat anti-rabbit- HRP [1:5,000 (Abcam)] for 2 h at room temperature. Image acquisition and densitometry analysis were performed using Image Lab software version 5.2.1 build 11 (Bio-Rad).

### Statistical analysis

Normality test was performed by Kolmogorov-Smirnov. Data are expressed by mean ± standard deviation (SD) for parametric values, while median and interquartile range (IQR) for non-parametric. *p < 0.05 and **p < 0.01 were considered statistically different. The comparison of litter size, sex ratio, mother, fetuses and placenta weights, and steroid hormones concentration between MO and C groups by Welch’s *t*-test. Pearson’s test was used to analyze correlations among maternal, fetal and placental weights, and hormones concentration. Since there were no differences in fetal weight between male and female per group, we have done correlations using averaged data.

The resulting melting curves after MS-HRM assays were converted to negative derivate peaks, for which the negative derivative of the fluorescence over the derivative of temperature (−dF/dT) is plotted against the increasing temperature. Heterogeneous methylation can only be estimated in a qualitative manner, for which the melting peaks in the highest melting temperature mean high methylation while melting peaks in the lowest temperature mean low methylation (Hussmann et al., 2018). The number of methylated CpG dinucleotide (mCpG) was accounted for directly Sanger sequencing results in both alleles (forward: F and reverse: R), and the average of CpGs between both alleles was determined.

RXRα mRNA and protein expression in umbilical cord were compared by nested *t*-test and two-way ANOVA. Analysis of correlation between RXRα mRNA and protein in umbilicar cord were performed using Spearman’s rank correlation test (r). Analysis and plots were performed using GraphPad Prism (version 9.2.0).

## Results

### Maternal, fetal, and placenta weights

**FIGURE 2:**
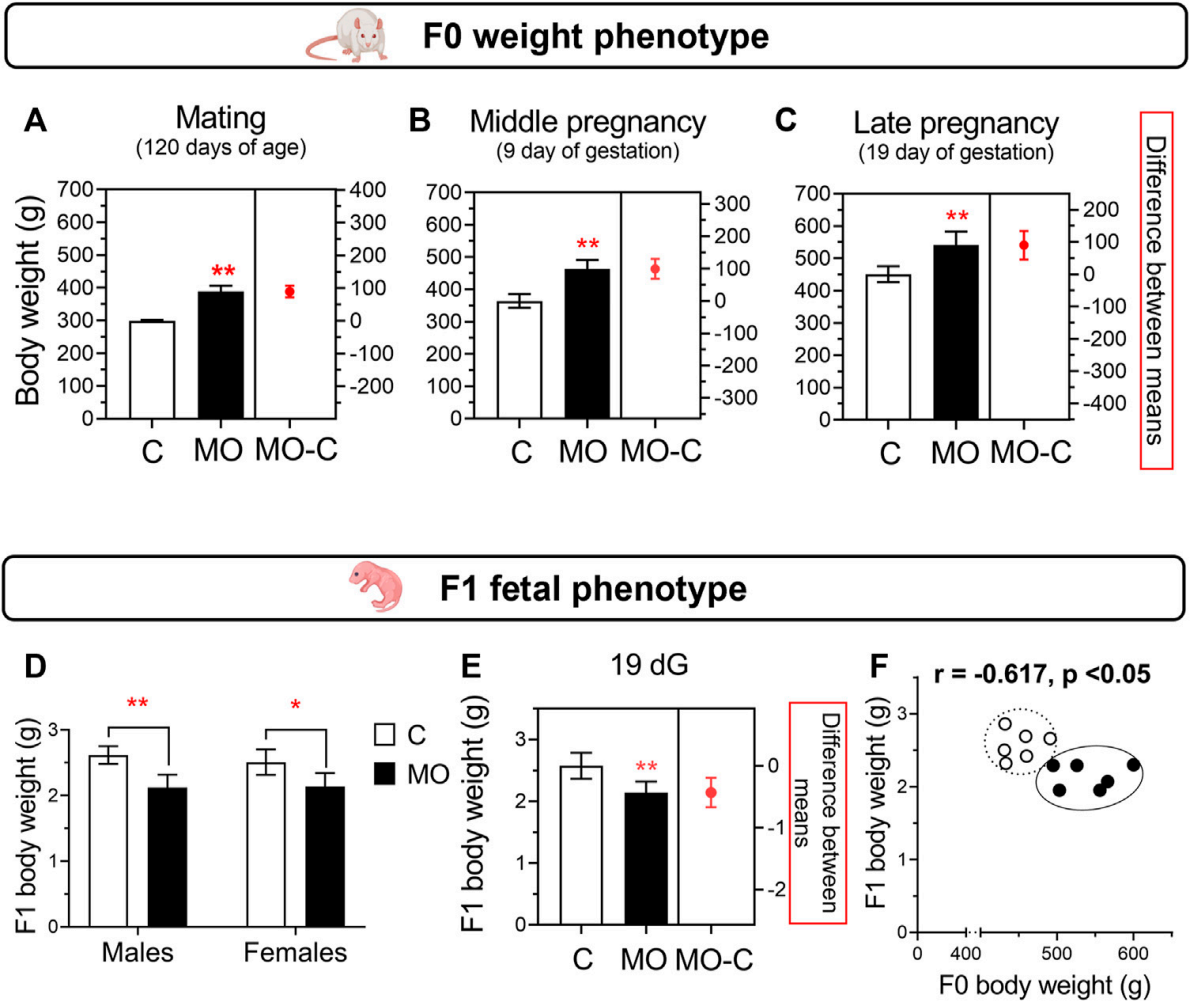
Maternal and fetal weight phenotypes. F0 body weight at mating **(A)**, middle pregnancy **(B)**, and late pregnancy **(C)**. Fetal body weight **(D)** and sex-averaged fetal weight **(E)** at 19 dG. Correlation between maternal and sex-averaged fetal weights **(F)**. Mean ± SD in bar plots (*n* = 6/group); *p < 0.05 and **p < 0.01 vs. C.

The MO group had similar body weight since 21 d (*C* = 51.4 ± 0.9 and MO = 51.7 ± 0.9 g, *p* > 0.05) until 63 d (*C* = 231 ± 3.1 and MO = 264 ± 5.5 g, *p* < 0.05), in which MO had an increased body weight compared to C until the end of the experiment (Figures 2A–C). During gestation, cumulative and total weight gain (*C* = 124 ± 12.5 and MO = 112 ± 20 g, *p* > 0.05) were similar between C and MO groups. Using averaged data, during gestation food intake per day was lower in MO than C group (*C* = 26.2 ± 3.5 and MO = 21.5 ± 1.8 g/day, *p* < 0.05) but energy intake was higher in MO than C (*C* = 89.2 ± 11.8 and MO = 103.2 ± 8.8 Kcal/day, *p* < 0.05). The energy intake per gram of body weight gained was similar between C and MO (*C* = 4,311 ± 2,597 and MO = 4,790 ± 2,772 Kcal/g, *p* > 0.05).

Litter size were similar between C and MO (*C* = 14.5 ± 1.8 and MO = 14.2 ± 4.0 pups/litter; *p* > 0.05). There were no differences in male:female ratio (*C* = 1.1 and MO = 1.0; *p* > 0.05). At 19 dG, placenta and body weight were similar between male and female for both groups. There were no differences between C and MO placenta weight (*C* = 0.62 ± 0.07 and MO 0.55 ± 0.11 g; *p* > 0.05). Total fetal weight in the litter was higher in C vs. MO (*C* = 37.4 ± 5.8 and MO = 29.7 ± 6.1 g, *p* < 0.05; Figures 2D,E); total placenta weight in the litter was similar between C and MO (*C* = 6.37 ± 3.8 and MO = 7.4 ± 1.0 g, *p* = 0.052). Total mass (fetal and placenta) per litter were similar among groups (*C* = 46.7 ± 7.5 and MO = 37.5 ± 7.0 g, *p* = 0.053). There is a negative correlation between maternal and fetal weight (Figure 2F), but no correlation between maternal and placental weight (*r* = −0.115, *p* > 0.05).

### Maternal serum steroid hormones and their correlation with maternal and fetal weights

**FIGURE 3:**
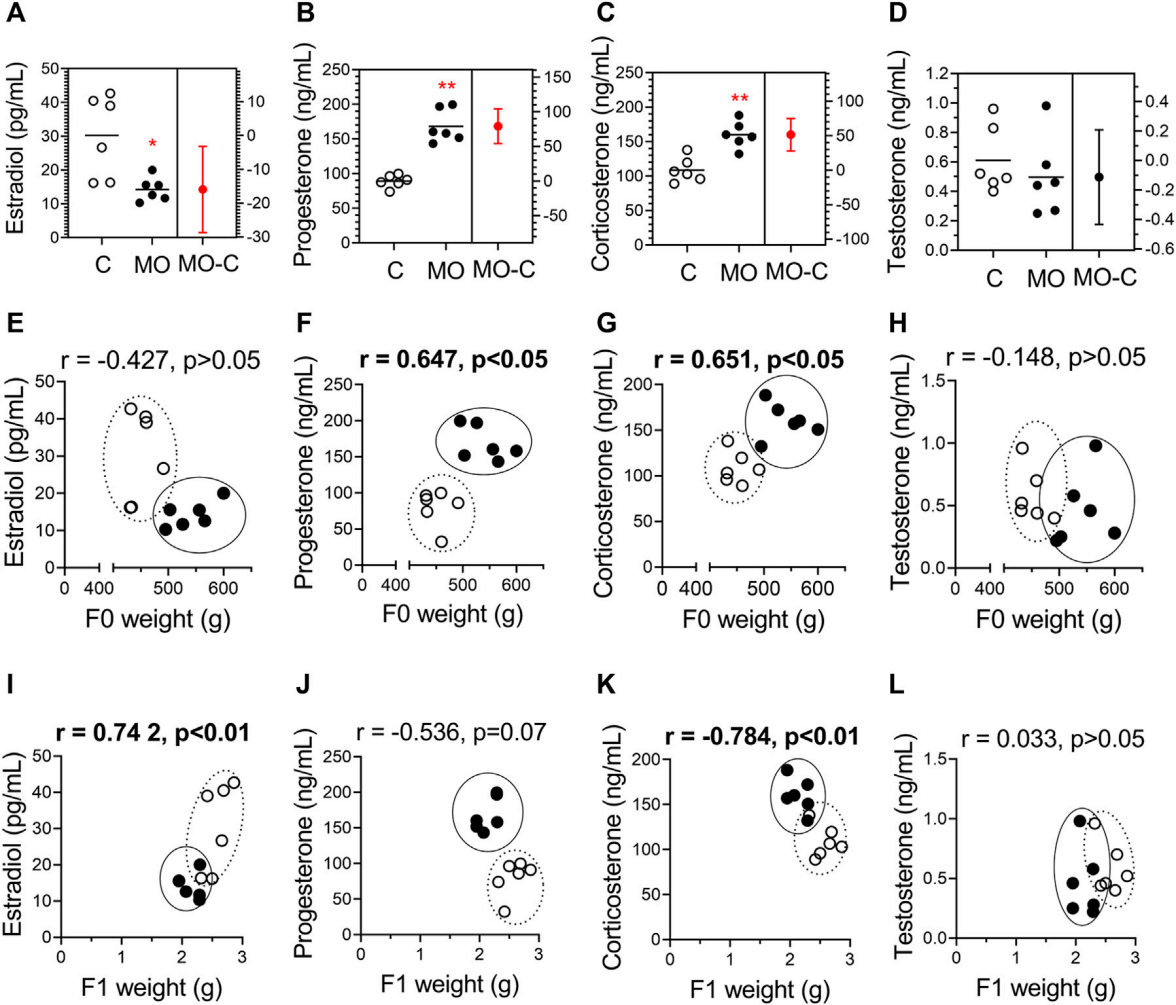
Stereoid hormones and their correlation with maternal and fetal weights. Maternal serum concentrations of estradiol **(A)**, progesterone **(B)**, corticosterone **(C)**, and testosterone **(D)** at 19 dG. Mean ± SD (*n* = 6/group); *p < 0.05 and **p < 0.01 vs. **(C)**. Pearson correlations (r) between hormones and maternal weight **(E–H)** and fetal weight **(I-L)**; *C* = white circles, MO = black circles.

Progesterone and corticosterone maternal serum concentrations were higher, and estradiol lower in MO group compared to C group. Testosterone maternal serum concentrations were similar between C and MO groups (Figures 3A–D). Positive correlations were found between progesterone and corticosterone with maternal weight, and estradiol with fetal weight, while negative correlation were found between corticosterone and fetal weight. No correlation were found between testosterone and maternal and fetal weights (Figures 3E–L).

### RXRα gene methylation status near early growth response-1 binding site

**FIGURE 4:**
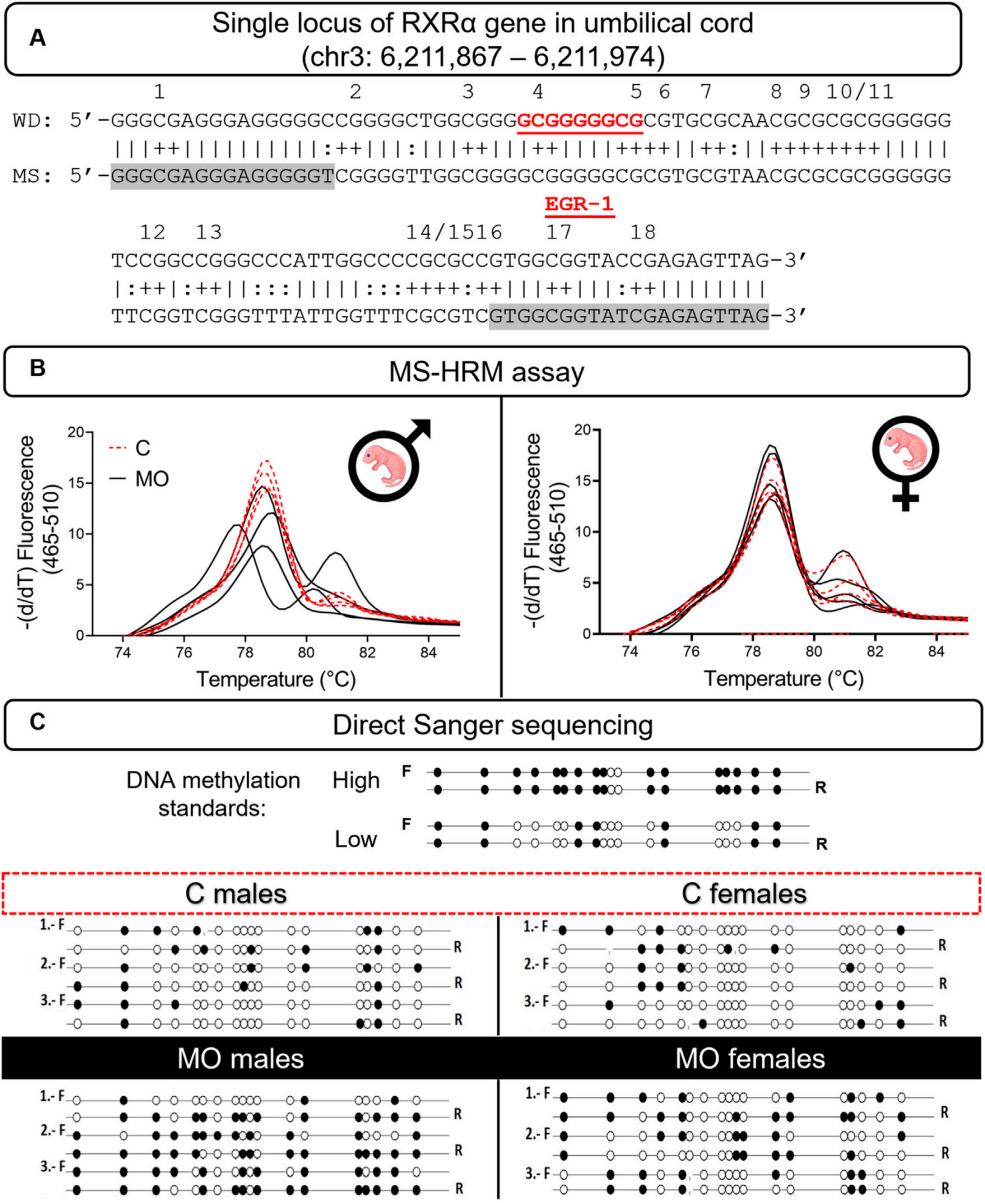
DNA methylation status of RXRα gene in offspring’s umbilical cord. RXRα sequence in a single locus of the chromosome 3 in the rat genome. DNA-binding site of EGR-1 transcriptional factor (red letters); primers sequence (highlighted in grey); CpG sites—numbers over sequence **(A)**. Melting peaks plots (*n* = 6/group/sex) show heterogeneuously methylation **(B)**. Lollipop diagram shows the validation of heterogeneuously methylation and individual mCpGs **(C)**. C, control; F, forward sequence; mCpGs, methylated CpG dinucleotides; MS, methylated sequence; MO, maternal obesity; R, reverse sequence; WD, wild sequence.

In the locus analyzed (Figure 4A), we observed an increased heterogeneous methylation state of the RXRα gene in the umbilical cord of MO fetuses in comparison to their controls. Furthermore, DNA methylation status of the RXRα gene in the umbilical cord of male MO fetuses was higher than in female MO fetuses (Figure 4B).

### Validation of RXRα gene methylation status

**FIGURE 5:**
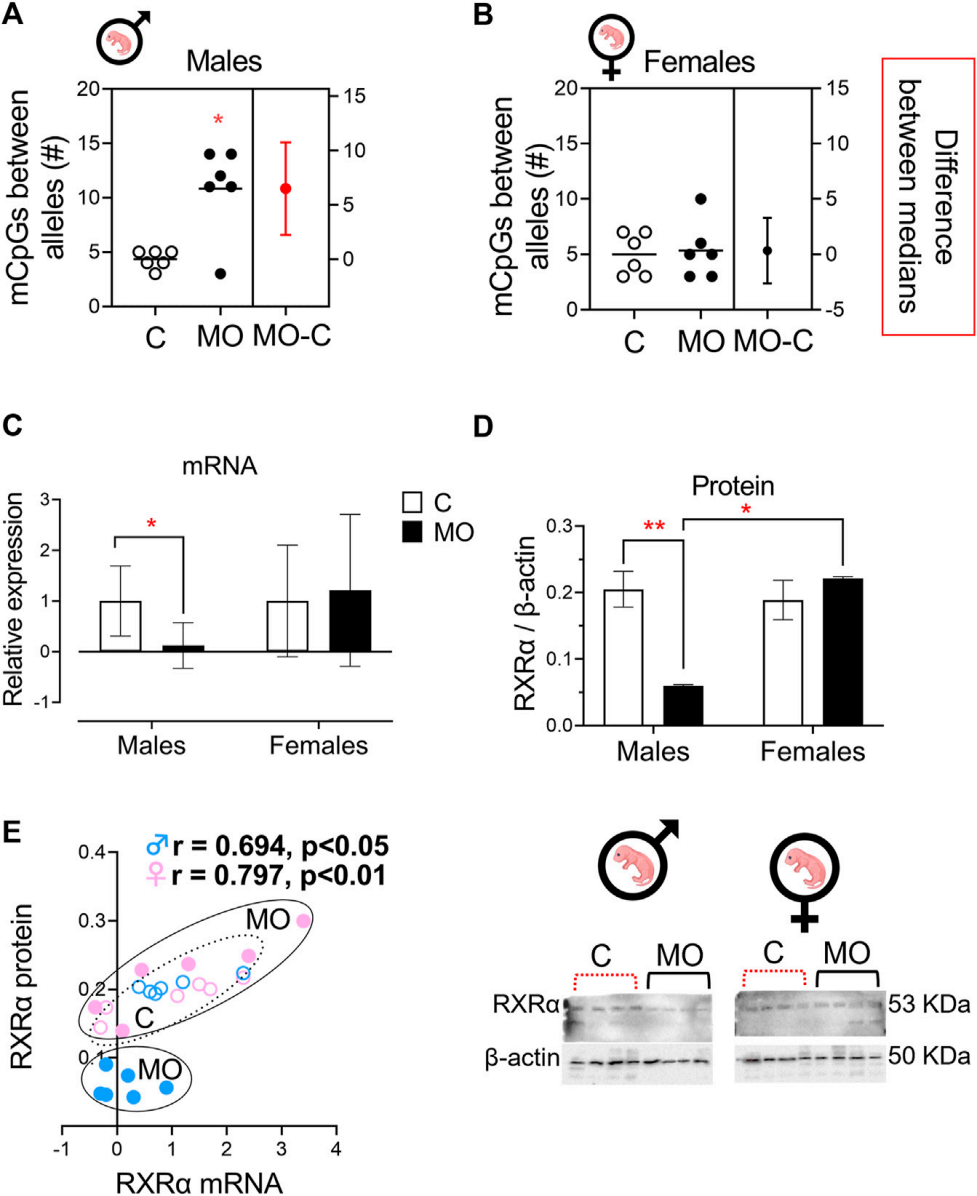
Allele-specific methylation and effects on RXRα mRNA and protein expression. Median with IQR. The average number of mCpGs in each allele (*n* = 3/group/sex) in umbilical cord of males **(A)** and females **(B)** offspring. Relative RXRα mRNA **(C)** and RXRα protein expression **(D)** (*n* = 6/group/sex). A representative western blot is showed below RXRα protein plot (*n* = 4/group/sex). Spearman correlation between RXRα mRNA and protein expression **(E)**. *p < 0.05 and **p < 0.001 vs. C.

MS-HRM products were sequenced for identifying specific sites of mCpGs between forward and reverse alleles in the examined sequence and to validate previous results. Our findings corroborated a high state of heterogeneous methylation composed of a range of 61%–78% mCpGs between alleles in the cord RXRα gene of male MO fetuses, while in female MO fetuses, the high state of heterogeneous methylation was composed of 28%–56% mCpGs between alleles. Respective controls of male and female fetuses showed a state of umbilical cord RXRα gene methylation about 17%–28% of mCpGs (Figure 4C). Finally, the total of mCpGs between alleles was higher in male MO fetuses than male C fetuses, and no differences were found between female MO and C fetuses (Figures 5A,B).

### Expression of RXRα mRNA and RXRα protein

At 19dG, RXRα mRNA and protein expression in the male MO umbilical cord was lower compared to C; no differences were found in the female groups (Figures 5C,D). There is a positive correlation between RXRα relative mRNA and protein expression for both male and female groups (Figure 5E).

## Discussion

Maternal obesity induces epigenetic changes in the offspring from fetal life onward that lead to metabolic problems including obesity in adulthood (Parlee and MacDougald, 2014; Rodríguez-González et al., 2019). Most of the experiments to study developmental programming due to maternal obesity and western diets have been conducted in rodents at different periods of maternal exposure to obesogenic diets and offspring developmental windows (pregnancy and/or lactation) (Bellisario et al., 2015; Rodríguez-González et al., 2015). It is important to consider the precise dietary components (macro- and micronutrients), food intake and extent and duration of MO and maternal high fat or sugar (or both) dietary intake before conception and during gestation. In this study we used the pregnant obese rat fed with high fat diet before and during pregnancy as experimental animal model to study maternal steroid hormones concentration, RXRα gene methylation near the EGR-1 binding site in the umbilical cord, as well as placenta and fetal weight at the end of gestation (19 dG).

Steroid hormones play an important role during pregnancy in developmental programming. In humans and rodents, maternal obesity during pregnancy has been associated with alterations of progesterone, estradiol, testosterone, and glucocorticoids (Rodriguez -Gonzailez et al., 2015; Vega et al., 2015; Maliqueo et al., 2017; Rodríguez-González et al., 2019). Circulating adrenal steroid hormones are small lipophilic molecules that regulate gene expression in a great variety of tissues, including the uterus, endometrium, and umbilical cord (Travers et al., 2018). These molecules bind to cognate receptors that exert post-translational modifications through interactions and nuclear translocations with cell and tissue-specific co-regulators (Houshdaran et al., 2020). Adrenal steroid hormones exert most of their physiological and regulatory functions of metabolic homeostasis through the binding and activation of orphan nuclear hormone receptors (Evans and Mangelsdorf, 2014), as is the case of RXRα protein.

Here, we investigated the maternal serum concentration of steroid hormones of F0 at 19 dG, resulting in increased progesterone and corticosterone and a decreased estradiol in the MO group compared to C, without changes in testosterone. The increased concentration of progesterone and the decreased concentration of estradiol in F0 MO group could be associated with the control of maternal body weight homeostasis to accommodate the fat deposition required to support fetal development and lactation, as well as deleterious maternal and placental functions (Zambrano et al., 2005; Lappas et al., 2020). The increased concentration of corticosterone in F0 MO group is consistent with our previous data before gestation and at the end of lactation, confirming that high serum concentrations of this glucocorticoid are a common featuring of response to stress generated by maternal obesity that repercusses on developmental programming (Zambrano et al., 2015; Rodríguez-González et al., 2019). One study in mice showed the increase of corticosterone concentration in obese mothers, maternal stressful challenge during pregnancy by high-fat diet comsumption, decreased placenta activity of 11beta-dehydrogenase-2 which implies that the protection to prevent the transplacental passage of surplus corticosterone is diminished, some of the consequences were the impairing neuroendocrine system and neural activity in the offspring (Bellisario et al., 2015). Although the adrenal gland is responsible for corticosterone production in the rat, the enzyme 11 beta-hydro steroid dehydrogenase—1 reductase in the adipose tissue can biotransform dehydrocorticosterone into corticosterone (Tomlinson et al., 2004) whereby adipose tissue can increase corticosterone concentration in the obese mother and be in part responsible of the accumulation of adipose tissue in the offspring in adult life.

DNA methylation is an epigenetic mechanism resulting in modulation of gene expression, mainly mediated by the control of transcription factors binding sites. Among other genes, specific DNA methylation marks have been identified in the RXRα gene promoter in newborns, and it has been associated with later adiposity in school-age children (Godfrey et al., 2011). Previous work in humans in our lab showed variability in DNA methylation of the RXRα gene promoter containing elements of response for EGR proteins in newborn’s cord blood, indicating the presence of diverse expression of this gene (Chávez-Lizárraga et al., 2018). Using the rat model, we confirmed the presence of heterogeneous RXRα gene methylation near the EGR-1 binding site in the fetus’s umbilical cord, showing that male fetal offspring exposed to maternal obesity had hypermethylation in the RXRα gene near the EGR-1 binding site. Experimental evidence supports that EGR-1 protein is a mediator for lipotoxicity-induced cytokine gene expression in the placentas from pregnant women with obesity, suggesting a relevant contribution to the effects of obesity on fetal programming (Saben et al., 2013).

A single locus of RXRα gene promoter methylation in umbilical cord has been associated with fat phenotypes of the offspring during childhood (Godfrey et al., 2011; Harvey et al., 2014). Moreover, experimental evidence in the Tet1 knockout mice showed that RXRα gene expression and RXRα protein activity are crucial for adipogenesis and adipocyte differentiation through DNA demethylation (Qian et al., 2021), showing that RXRα plays a critical role in adipogenesis. Previously, we have shown in rat offspring from obese mothers, fat tissue expansion (Ibáñez et al., 2018) and premature metabolism aging (Rodríguez-González et al., 2019) in a sex dependent manner.

Our data in umbilical cord are in accordance with other studies that showed DNA methylation of the RXRα gene as one of the main epigenetic mechanisms implied in the regulation of RXRα mRNA and RXRα protein expressions in fetal organs/cells as the placenta and trophoblasts (Pospechova et al., 2009; Nakanishi et al., 2021). DNA methylation of RXRα in the placenta correlates inversely to RXRα mRNA expression linked to maternal choline consumption, birthweight and body mass index in humans (Nakanishi et al., 2021). Our evidence of distinct RXRα mRNA and protein expressions between umbilical cords of male and female fetuses exposed to high-fat diets supports the hypothesis about a sex-specific response based on RXRα regulation linked to lipid metabolism in rodent tissues (Kosters et al., 2013) and the endocrine system (Sarr et al., 2012).

Findings discussed here thus far support the idea that maternal obesity and high-fat diet during pregnancy contributes to the epigenetic programming and explains a potential mechanism to develop lifelong metabolic problems in the offspring. Further exploration of mechanisms involved in the sexual dimorphism of metabolic imprinting is needed to propose targeted interventions that have an impact on the offspring life quality.

**FIGURE 6:**
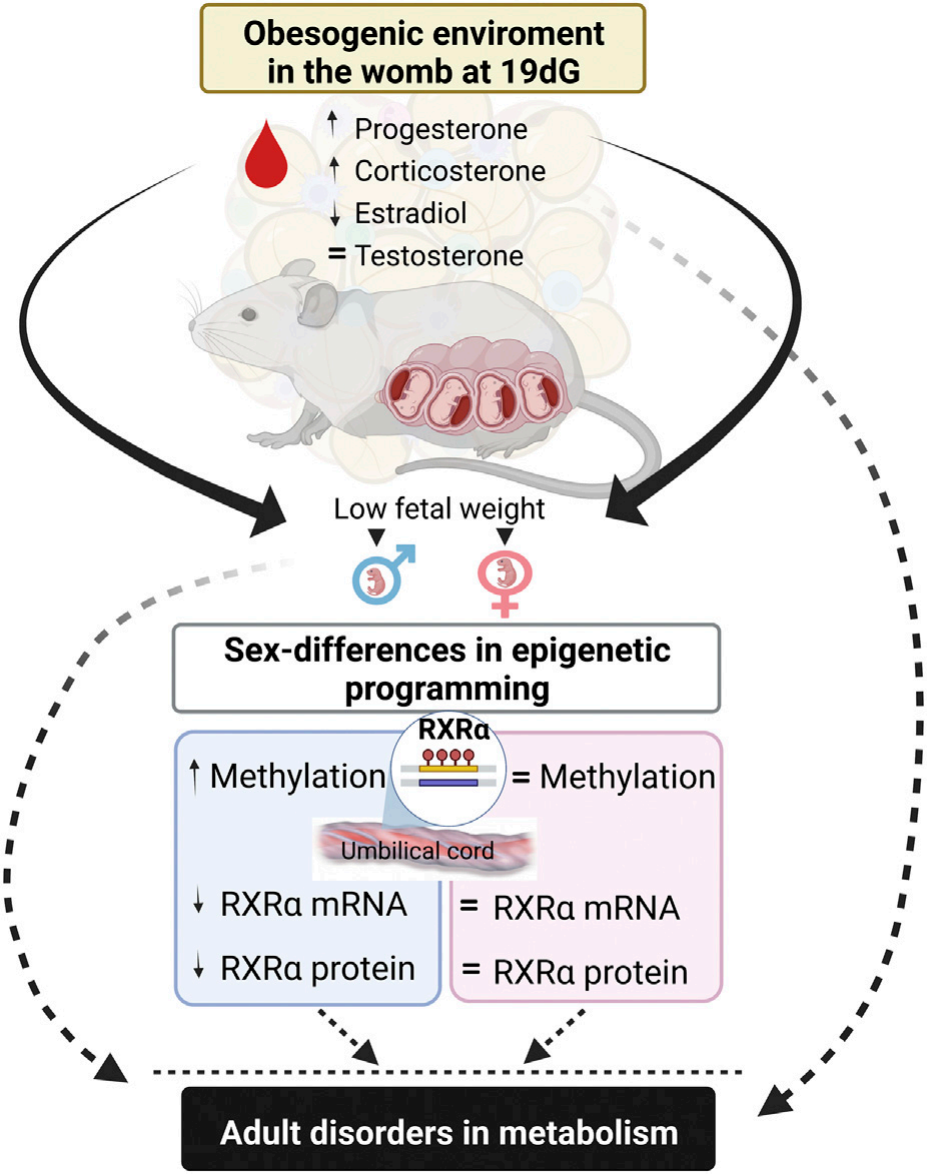
Summary of findings. Maternal high-fat diet modify stereoid hormone concentrations that correlates with low fetal weight and promotes umbilical cord hypermethylation in RXRα gene and decrases mRNA and protein expressions in a sex-dependent manner. These findings explain potential mechanism of offspring metabolic programming by maternal obesity.

In summary, maternal obesity increases corticosterone and decreased estradiol serum levels at the end of gestation, which correlates with lower fetal weight. Interestingly, we observed male umbilical cord hypermethylation of the RXRα gene, with no changes in females, showing sexual dimorphism in fetal stages. These findings help to explain potential mechanisms of maternal obesity in the metabolic programming of the offspring (Figure 6).

In conclusion, serum steroid hormones changes in the obese mothers negative impacts fetal weight; obesogenic environment induces epigenetic changes of RXRα methylation in umbilical cord in a sex-specific manner. These findings show a potential mechanism explaining the association between maternal obesity with adipose tissue dysregulation, impaired metabolism and lifelong obesity in the offspring.

## Data Availability

The original contributions presented in the study are included in the article. Further inquiries can be directed to the corresponding author.
